# Evidence-based Recovery Colleges: developing a typology based on organisational characteristics, fidelity and funding

**DOI:** 10.1007/s00127-023-02452-w

**Published:** 2023-03-11

**Authors:** Daniel Hayes, Elizabeth M. Camacho, Amy Ronaldson, Katy Stepanian, Merly McPhilbin, Rachel A. Elliott, Julie Repper, Simon Bishop, Vicky Stergiopoulos, Lisa Brophy, Kirsty Giles, Sarah Trickett, Stella Lawrence, Gary Winship, Sara Meddings, Ioannis Bakolis, Claire Henderson, Mike Slade

**Affiliations:** 1 Health Service and Population Research Department, Institute of Psychiatry, Psychology and Neuroscience, King’s College London, De Crespigny Park, London, SE5 8AF UK; 2 Department of Behavioural Science and Health, University College London, London, WC1E 7HB UK; 3 Division of Population Health, Health Services Research and Primary Care, School of Health Sciences, Faculty of Biology, Medicine and Health, Manchester Centre for Health Economics, The University of Manchester, Oxford Road, Manchester, M13 9PL UK; 4 School of Health Sciences, Institute of Mental Health, University of Nottingham, Nottingham, NG7 2TU UK; 5 ImROC, Nottinghamshire Healthcare NHS Foundation Trust, Duncan Macmillan House, Porchester Road, Mapperley, Nottingham, NG3 6AA UK; 6 Nottingham University Business School, Nottingham, NG8 1BB UK; 7 Centre for Addiction and Mental Health, Department of Psychiatry, University of Toronto, Toronto, ON M5T 1R8 Canada; 8 School of Allied Health, Human Services and Sport, La Trobe University, Melbourne, VIC Australia; 9 South London and Maudsley NHS Foundation Trust, Denmark Hill, London, SE5 8AZ UK; 10 RECOLLECT Lived Experience Advisory Panel, Nottingham, UK; 11 School of Education, University of Nottingham, Nottingham, NG2 5BY UK; 12 Nord University, Postboks 474, 7801 Namsos, Norway

**Keywords:** Recovery College, Survey, Managers, England, Mental health services, Service costs

## Abstract

**Purpose:**

Recovery Colleges (RCs) have been implemented across England with wide variation in organisational characteristics. The purpose of this study is to describe RCs across England in terms of organisational and student characteristics, fidelity and annual spending, to generate a RC typology based on characteristics and to explore the relationship between characteristics and fidelity.

**Methods:**

All RC in England meeting criteria on recovery orientation, coproduction and adult learning were included. Managers completed a survey capturing characteristics, fidelity and budget. Hierarchical cluster analysis was conducted to identify common groupings and generate an RC typology.

**Results:**

Participants comprised 63 (72%) of 88 RC in England. Fidelity scores were high (median 11, IQR 9–13). Both NHS and strengths-focussed RCs were associated with higher fidelity. The median annual budget was £200,000 (IQR £127,000–£300,000) per RC. The median cost per student was £518 (IQR £275–£840), cost per course designed was £5,556 (IQR £3,000–£9,416) and per course run was £1,510 (IQR £682–£3,030). The total annual budget across England for RCs is an estimated £17.6 m including £13.4 m from NHS budgets, with 11,000 courses delivered to 45,500 students.

**Conclusion:**

Although the majority of RCs had high levels of fidelity, there were sufficiently pronounced differences in other key characteristics to generate a typology of RCs. This typology might prove important for understanding student outcomes and how they are achieved and for commissioning decisions. Staffing and co-producing new courses are key drivers of spending. The estimated budget for RCs was less than 1% of NHS mental health spending.

**Supplementary Information:**

The online version contains supplementary material available at 10.1007/s00127-023-02452-w.

## Background

Recovery Colleges (RCs) are a relatively new approach to supporting individuals with their mental health [1]. Starting in the UK in 2009, and adapted from the concept of Recovery Education Centres that were pioneered in the US in the 1990s as a way to support individuals with mental health issues and address health inequalities, RCs now exist in over 20 countries [2]. International adoption has been catalysed by the recovery movement which is now a cornerstone of many countries healthcare policy [3–6], including the UK [7] where it has gained widespread momentum [8, 9].

Key principles for RCs include a focus on supporting personal recovery through adult education and co-production [10]. Supporting recovery involves enabling students to experience key recovery processes of connectedness, hope, identity, empowerment, meaning and purpose [11], and incorporates clinical, societal and personal recovery aspects [12]. An adult education approach involves students and trainers sharing experiences, knowledge and skills to develop students’ strengths through participation in courses which facilitate the learning of new skills to self-manage recovery [13]. Co-production involves people with lived experience working alongside other experts to design and deliver all aspects of the RC [13]. These key principles and components can be assessed using the RECOLLECT Fidelity Measure [10].

Models and conceptual frameworks of RCs suggest that they may not only benefit students, but staff and wider society as well [14, 15]. For students, RCs are thought to work by shifting the balance of power, enabling new relationships to form between stakeholders and facilitating personal growth, resulting in both changes to the student and their wider life [15]. For staff, interactions with students positively impact on relationships and change attitudes to professional practice via coproduction [14]. At a societal level, RCs provide opportunities for members of the public to learn alongside, and community organisations to work with, those with mental health issues [15].

A recent review on the evidence base for RCs identified that they were associated with many benefits, including attainment of recovery goals, improved quality of life and well-being, increased knowledge and self-management skills, reduced service use and changes in service providers’ practice [16]. However, little information was identified on the costs associated with RCs, with only two studies in England reporting on this [17, 18]. Organisational characteristics were also not reported despite there being variation in how RCs operate and run and aspects of fidelity could not be measured due to the RECOLLECT Fidelity Measure having only recently been developed [10].

An understanding of organisational and student characteristics, fidelity and running costs, is, therefore, needed to inform future research into their outcomes and how they are achieved, as well as future commissioning arrangements.

## Objective

To describe organisational and student characteristics, fidelity and funding of RCs in England; to examine relationships between organisational characteristics and fidelity; and to investigate whether specific types of RCs could be grouped based on shared characteristics.

## Methods

This study was conducted as part of the National Institute for Health Research (NIHR) programme grant ‘Recovery Colleges Characterisation and Testing (RECOLLECT) 2’ (NIHR200605; researchintorecovery.com/recollect).

### Design

Closed online national survey.

### Setting

All RCs in England.

### Participants

Inclusion criteria, as rated by the manager and using an established approach [10] with definitions as given above and operationalised in Supplementary material 1, were that the RC:

- focuses on supporting personal recovery
- aspires to use co-production
- aspires to use adult learning approaches
- is physically based in England

### Measures

The survey is shown in full in Supplementary material 1. In addition to questions establishing eligibility, it contained the RECOLLECT Fidelity Measure and questions about organisational, student and funding characteristics.

The RECOLLECT Fidelity Measure is a 12-item RC manager-rated measure (contained in Supplementary material 1) assessing seven ordinal and five categorical components of a RC [10]. The seven ordinal components are each scored from 0 (low fidelity) to 2 (high fidelity): Valuing equality; Learning; Tailored to the student; Co-production; Social connectedness; Community focus; Commitment to recovery. The fidelity score is the sum of these seven items, ranging from 0 (low fidelity) to 14. The five Categorical components are rated as either Type 1 or Type 2: Available to all [Anyone versus Specific groups] (anyone from the local community versus just e.g. mental health service users, carers and staff); Location [Community versus Statutory services]; Distinctiveness of course content [Mainstream versus Non-mainstream]; Strengths-based [Implicit versus Explicit]; and Progressive [No goal-setting versus Goal setting] (whether goal-setting is not an explicit focus versus use of specific goal-setting approaches such as Individual Learning Plans). No summary score is calculated for categorical items, since their relationship with outcome has not been investigated. Psychometric evaluation involving 39 RCs in England showed that the measure meets scaling assumptions and demonstrates adequate internal consistency (0.72), test–retest reliability (0.60), content validity and discriminant validity [10].

### Organisational and student characteristics

Questions were developed by integrating the RECOLLECT change model [15]; which explores the impact on RCs on student outcomes, and the RECOLLECT multilevel change model [14] which explores the impact of RCs on staff and societal outcomes with previous RC characteristic surveys [2, 19] and established frameworks for describing interventions [20, 21].

### Funding characteristics

Economic information focussed on budget (total annual budget and key categories: staff, rent and technology), staffing profiles (paid and voluntary roles), funding sources, business cases submitted for large spending and the impact of the COVID-19 pandemic on budgets and spending.

### Procedures

Five approaches were used to identify potentially eligible RCs in England: (a) web searches (‘Recovery college’ OR ‘Discovery Centre’ OR ‘Empowerment College’ OR ‘Wellbeing College’ OR ‘Recovery Academ*’); (b) expert consultation with RC national leaders; (c) existing recovery networks such as ImROC (imroc.org) and the Recovery Research Network (researchintorecovery.com/rrn); (d) snowball sampling with participating RC managers and (e) telephone calls to potential host charities and mental health NHS Trusts (mental health service provider organisations). The search was conducted in June and July 2021.

The research team contacted all identified organisations, using email, phone and social media platforms, to ascertain if the service met inclusion criteria as a RC, with up to three further follow-up contacts where needed. Non-responding organisations were discussed with identified RC managers and national leaders to ascertain whether they were still operating. Those known to be operating were included in the final sample, whilst those understood to be no longer operating were excluded.

A pilot version of the developed survey informed by the CHERRIES guidelines [22] was implemented on Qualtrics (www.qualtrics.com) and then refined by expert review (study team and a clinician with experience of working at a RC) and piloting by two RC managers. The final survey is shown in Supplementary material 1. As this was a closed survey accessible only through personal log-in, no cookies, IP check or log file/timestamp analysis were used.

The survey opened in August 2021 and closed in October 2021. All responding organisations which met RC inclusion criteria were contacted and asked to complete the survey. A minimum of three reminders, mainly emails, were sent to encourage completion. Completeness checking was conducted after submission so as not to disincentivise partial completion, with further liaison with the responding RC where indicated. Data from partially completed surveys were included. No financial incentives for participation were offered.

The authors assert that all procedures contributing to this work comply with the ethical standards of the relevant national and institutional committees on human experimentation and with the Helsinki Declaration of 1975, as revised in 2008. All procedures involving human subjects/patients were reviewed by Nottinghamshire Healthcare NHS Foundation Trust as Sponsor and deemed not to need research ethics committee approval as the survey was about usual practice with no patient-level data collected. Nonetheless, electronic informed consent was obtained from all RC manager participants prior to survey completion.

### Statistical analyses

To explore the relationship between organisational characteristics and fidelity, we used independent *t*-tests to compare the total fidelity score with each categorical component.

To identify types of RC, cluster analysis was performed. Variables (*n* = 16) relevant to characterising RCs were identified a priori from the RECOLLECT change and multilevel change models [10, 14] and through consultation with the RECOLLECT Lived Experience Advisory Panel (LEAP), a panel of 10 advisors to the study with a range of relevant lived experience, and the RECOLLECT International Advisory Board (*n* = 11) of international experts. Included variables comprised: years of operation; location (urban, suburban, rural, mixed); total students per year; organisational affiliation (NHS affiliated versus non-NHS affiliated), and all 12 items from the RECOLLECT Fidelity Measure [10]. We used agglomerative hierarchical cluster analysis with a weighted average linkage and Gower’s distance applied due to the mixed nature of the variables included (i.e. continuous, binary and categorical). The optimal number of clusters was determined by inspection of the dendrogram and confirmed using the Calinski–Harabasz index (elbow method) and Duda–Hart index. Distinctions between clusters on outcome variables of interest were examined using analysis of variance (ANOVA) for continuous variables and Chi-square tests for binary and categorical variables.

To explore economic data pertaining to budgets, staffing, funding sources and pandemic spending, summary statistics were generated. For each summary statistic, all RCs with available data were included. Costs are reported in 2021/22 pounds sterling (£). Key unit costs (cost per student; cost per course designed; and cost per course delivered—courses were designed once but often delivered multiple times) were calculated by dividing the annual budget reported by each RC by the number of students and courses. To estimate annual RC spending in England, the mean and median costs, student numbers and course numbers per RC were multiplied by the number of RCs identified in our mapping exercise. The proportion of funding which RCs reported receiving from the NHS was applied to the nationwide cost to estimate the annual NHS spending on RCs.

All analyses were conducted using STATA 17.0 [23].

## Findings

Our national search identified 134 potential RCs, of which 88 (66%) reported meeting our inclusion criteria. The most common reasons for exclusion were non-contactable and deemed no longer operational in conjunction with national experts (*n* = 20), duplicate name (*n* = 6), satellite site of included RCs (*n* = 5) and not currently running (*n* = 5). Full details of reasons for exclusion of all 46 organisations are shown in Supplementary material 2.

### Recovery College characteristics

Overall, 63 (72%) of the 88 identified RCs participated, comprising 54 with full data and 9 with partial data completion, primarily missing economic data. The organisational characteristics of participating RCs are shown in Table 1.

**Table 1:** Organisational characteristics of Recovery Colleges (*n* = 63)

Organisational characteristic	Mean ± SD, *N* (%)
Time in operation (years)	5.6 ± 2.3 Range 0–10
Location	
Urban	21 (33)
Suburban	5 (8)
Rural	2 (3)
Mixed urban/suburban/rural	35 (56)
Fixed single-use physical base	
Yes	32 (51)
No—meet in community or mixed-use venues	30 (48)
No—virtual college operating only online	1 (2)
Main organisational affiliation	
Statutory health service, e.g. NHS Trust	43 (68)
Non-governmental organisation	19 (30)
Local authority, e.g. council	5 (8)
Independent	3 (5)
Other health, e.g. private healthcare provider	2 (3)
Education provider, e.g. university or college	2 (3)
Other	1 (2)
Leadership team includes people with mental health lived experience?	
Yes	58 (92)
No	5 (8)
Groups most commonly involved in co-production?	
Lived experience + health or social care professional	45 (71)
Lived experience + community topic expert	12 (19)
Lived experience only	4 (6)
Other	2 (3)
More important goal of the Recovery College	
To reduce stigma and discrimination in society	5 (8)
To positively impact on MH services	1 (2)
Both equally important	57 (91)
Uses goal-oriented personal plans?	
Yes	30 (48)
No	33 (52)

Key findings are: the diversity in geographical setting, around half of RCs have a dedicated building, around two-thirds are NHS Trust-affiliated, most (92%) include lived experience leadership and co-production has a range of meanings.

## Student characteristics

Full student characteristics, including ethnicity, are shown in Supplementary material 3. Recovery Colleges reported having a median of 300 students per year (IQR 125–575). Students had a mean age of 40.7 (6.9) years with 57.5% (18.1) of being female. Forty-one (65%) of RCs reported being open to the public who may have no connection with the mental health system, whilst 58 (92%) reported being for people with mental health issues using secondary mental health services. Many RCs or RC campuses and satellite sites reported catering for specific groups, including 19 (30%) for students with substance use disorders, 18 (29%) for students who are unemployed, 17 (27%) for students in forensic services and 17 (27%) for Black and minority ethnic students.

## Fidelity

RECOLLECT Fidelity Measure scores are shown in Table 2.

**Table 2:** RECOLLECT Fidelity Measure item scores of Recovery Colleges (*n* = 63)

Ordinal components	Low (0) *N* (%)	Medium (1) *N* (%)	High (2) *N* (%)
Equality	1 (2)	12 (19)	50 (79)
Adult learning	3 (5)	30 (48)	30 (48)
Tailored to the student	0 (0)	31 (49)	32 (51)
Co-production	4 (6)	19 (30)	40 (64)
Social connectedness	9 (14)	27 (43)	27 (43)
Community focus	7 (11)	23 (37)	33 (52)
Commitment to recovery	2 (3)	17 (27)	44 (70)

Categorical components	Type 1 *N* (%)	Type 2 *N* (%)
Available to all	[Anyone] 44 (70)	[Specific groups] 19 (30)
Location	[Community] 30 (48)	[Statutory] 33 (52)
Distinctiveness of course content	[Mainstream] 27 (43)	[Not mainstream] 36 (57)
Strengths	[Implicit] 13 (31)	[Explicit] 50 (79)
Progressive	[No goal setting] 41 (65)	[Goal setting] 22 (35)

The median fidelity score was 11 (IQR 9–13). Half of RCs reported high scores on five of the seven ordinal components, with Equality the highest scoring component. For categorical components, 50 (79%) of RCs reported that a focus on strengths was made explicit within the RCs and 41 (65%) did not focus on goal setting.

Comparison of each categorical component with fidelity score is shown in Supplementary material 4. The location and strengths components showed significant differences. Recovery Colleges based in community locations which were not shared with health, social care or other statutory services scored higher on fidelity (11.6 (SD 1.9)) versus 9.8 (SD 2.8), *p* = 0.003)), as did RCs which focussed explicitly on strengths (11.1 (SD 2.3)) versus those that implicitly did so (9.1 (SD 3.0), *p* = 0.012)).

## Cluster analysis

To identify different types of RCs, we conducted a cluster analysis, reported in Supplementary material 5. Four clusters were observed within the dendrogram (Fig. S5.1) and confirmed using the Calinski–Harabasz (elbow method) and the Duda–Hart index. Cluster 1 contained 42 RCs, cluster 2 contained 17 RCs, cluster 3 contained three RCs and cluster 4 contained one RC and was deemed an outlier. Therefore, results supported a three-cluster solution.

Organisational characteristics (Table S5.1), student characteristics (Table S5.2) and fidelity scores (Table S5.3) were described for each cluster and differences between these factors were investigated. Clusters differed significantly on two organisational characteristics (location, main organisational affiliation), six student characteristics (% males, % females, catering for people with mental health issues who are using no services or only primary care or voluntary sector mental health services, catering for people with mental health issues who are using secondary mental health services, catering for informal carers of people with mental health issues and catering for forensic groups) and seven fidelity items (Equality, Social connectedness, Community focus, Commitment to recovery, Available to all, Location and Strengths). These informed our interpretation of clusters 1 to 3, shown in Table 3.

**Table 3:** Characteristics of Recovery Colleges (*n* = 62) by cluster

Cluster interpretation	*N*	Main characteristics
Strengths-oriented Recovery Colleges	42	• Largely in mixed (urban and suburban and rural) settings • Almost exclusively affiliated with an NHS trust • Mainly available for any member of the public to attend • Mainly based on health/social care service buildings • Strengths focus is mainly explicit
Community-oriented Recovery Colleges	17	• Almost exclusively NOT affiliated with an NHS trust • Mainly available for any member of the public to attend • Always based in a community location • Strong focus on social connectedness
Forensic Recovery Colleges	3	• Largely male students • Specific focus on forensic populations • Only available to specific groups, not to any member of the public • Strengths focus is always implicit

## Economic analysis

The budgets, number of students and number of courses are shown in Table 4.

**Table 4:** Overview of Recovery College budgets, students and courses

	*N*	Mean (SD)	Median (IQR)	Range
Annual budget	50	£233,227 (166,730)	£200,000 (£127,000–£300,000)	£15,000–£696,000
Number of students	63	517 (740)	300 (125–575)	50–4919
Number of courses (total)	62	197 (222)	125 (60–220)	20–1200
Number of individual courses	63	44 (50)	33 (25–45)	2–379
Number of courses taken per student	46	5.8 (3.9)	4 (3–6)	1–20
Cost per student	50	£877 (1,140)	£518 (£275–£840)	£10–£6,400
Cost per individual course designed	50	£8101 (9,663)	£5,556 (£3,000–£9,416)	£429–£50,000
Cost per course run	50	£2111 (2,061)	£1510 (£682–£3030)	£175–£10,300

The higher cost per course designed versus course run reflects the resource intensity of co-producing each course.

England-wide figures were estimated based on the 88 identified institutions meeting criteria for inclusion as a RC in the survey. Based on mean findings, £20.5 m is the England-wide spend on RCs, with 45,496 students attending, 3872 courses designed and 17,336 courses delivered. Based on median findings, £17.6 m is the England-wide spend on RCs, with 26,400 students attending, 2904 courses designed and 11,000 courses delivered.

Additional economic investigation of funding sources and spending is shown in Supplementary material 6 (S6.1–S6.7).

## Funding

From the 48 RCs that provided relevant data, it was estimated that 76% of funding received was from the NHS, either through NHS Trusts or CCGs, shown in Fig. S6.2. On the basis of the average annual costs for the 88 RCs in England, the NHS spends an estimated £13.4 m (based on median costs) to £15.6 m (based on mean costs) on RCs per year.

Overall, 35 RCs had 1 funding source, with funding sources comprising CCGs (*n* = 12 RCs), NHS Trusts (*n* = 15), charity (*n* = 3), local authority/council (*n* = 4) and self-funded (*n* = 1). A further 24 RCs had more than 1 funding source, outlined in Table S6.3.

Table S6.1 explored differences between the median budgets within clusters. Annual budgets were similar (£109,000 for a cluster 1 RC versus £206,000 cluster 2 versus £150,000 cluster 3), but cluster 3 differed in having fewer students (357 versus 300 versus 60) and greater cost per student (£504 versus £618 versus £2,308).

## Budgets

The largest proportion of RC budgets was for staff (mean 76%), followed by rent, technology and staff training, shown in Table S6.4. Pay scales for various RC roles were investigated in Table S6.5. The two largest core staff groups differ in pay: peer trainers are paid on average less (range: Bands 3–6) than non-peer healthcare professional trainers (range: Bands 4–7). Fifteen RCs reported having unpaid/voluntary roles which were core to operations, with a median of 28 h per week contribution. Median annual budget for these RCs was £105,000, compared with a median annual budget of £206,000 for RCs not using unpaid core roles.

Many RCs reported involvement of additional healthcare professionals in co-producing and co-facilitating, mostly funded by other budgets not attributed to the RC, investigated in Table S6.7. Amongst the 59 RCs who reported on these roles, the most common role was occupational therapist (24 (41%) RCs, median 45 h per year), followed by psychologists (23 (39%), 20 h), nurses (20 (34%), 30 h), allied health professionals (19 (32%), 22 h) and psychiatrists (11 (19%), 8 h).

In relation to COVID-19 (Table S6.9), 39 (65%) of RCs identified an impact of the pandemic on their spending, both with reduced venue hire costs arising from moving online and increased technology and online service costs.

## Conclusions

This national survey found 88 RCs were operating in England in 2021, with a median of 300 students. Sixty-five percent were open to the public who may have no connection with mental health services, whilst others catered to specific sociodemographic and clinical groups. RCs could be grouped into three distinct clusters: strengths-oriented, community-oriented and forensic. Most RCs scored high on fidelity; it appears that each of a strength and a community focus are associated with high fidelity. Extrapolating from participating RCs, around £20 m per year is spent on RCs, including around £15 m from NHS budgets. Many RCs have unpaid staff in core roles, and these RCs have a lower annual budget. Many RCs also involve healthcare professionals from a range of disciplines including occupational therapists, psychologists, nurses and psychiatrists.

## Organisational characteristics

The survey suggests a rapid expansion in the 12 years since the first RC opened in South West London in 2009 [13] and continued expansion since the last national survey identified 75 RCs in 2017 [19].

The dominant organisational affiliation for RCs was with statutory health services, which fits with recent shifts in national and international policy towards recovery-oriented care [24]. However, one-quarter of RCs was wholly or mainly funded from other sources, including local authorities and charities. This indicates that a mixed economy of care has emerged. The relationship between RC commissioning arrangements and student outcomes will be an important future research area.

## Recovery College operating characteristics

Almost all RCs reported lived experience on their senior leadership teams, a balanced focus on societal and mental health service impact, and high scores on ordinal fidelity components. This suggests that there is broad consensus about defining features of RCs. However, 71% of RCs operationalised co-production as meaning involvement from people with lived experience and from healthcare professionals, 19% as involvement from people with lived experience and from community topic expert involvement and 6% as only involving people with lived experience. This indicates that the defining feature of co-production is interpreted differently across the RC community. Given the concerns emerging from the survivor movement about the operationalisation of co-production in mental health services [25], the implications of these different approaches to co-production would benefit from investigation. The RECOLLECT Change Model proposed that key mechanisms of student benefit are an empowering environment, shifting the balance of power, enabling different relationships and facilitating personal growth [15]. The extent to which different operationalisations of co-production support different mechanisms of action could be explored through realist evaluation [26].

## Types of Recovery College

Three clusters of RCs were identified, which were interpreted as strengths-oriented, community-oriented and forensic. Although the forensic cluster comprised very few RCs, we believe that this cluster has some unique, important characteristics not seen in other clusters (i.e. largely male, forensic populations). Two long-standing criticisms of mental health services are that they are overly deficit-oriented [27] and that explanatory models de-contextualise mental health issues [28]. One explanation for the emergence of the first two clusters would be that the more NHS-based strengths-oriented RCs are prioritising addressing the first criticism through an explicit focus on strengths, and the non-NHS-based community-oriented RCs are prioritising addressing the second criticism through more support for social inclusion. The potentially differential impact of these three types of RCs on student outcomes is an important area and will be investigated as part of the RECOLLECT programme in the UK [29]. A typology of the recovery-oriented services in Australia ‘Prevention and Recovery Care’ similarly identified three clusters in a recent analysis [30]. However, these focussed almost exclusively on operational aspects (length of service, step down admissions, if families participated in meetings and quality Indicators for Rehabilitative Care (e.g. the buildings and where meetings take place)). Our findings suggest service values and ethos also play a crucial role in determining typology and should be captured and included.

## Economic considerations

Our analysis indicated two key drivers of cost. First, co-development of courses is resource-intensive, with each course costing around £8,000 to develop. Consideration might be given to whether RCs sharing courses could help reduce this cost. For example, some courses—such as ‘Introduction to recovery’ and ‘Telling your story’—are run across most RCs [13]. Whilst a strong emphasis exists within RCs about the importance of each course being co-produced locally, having course templates which could be locally tailored may help free up resource and time for other activities. The other advantages of sharing course content between RCs include quality control and sharing of pedagogical innovations.

By far, the largest proportion of RC budgets was allocated to staffing. Staffing profiles were complex and varied, hence we reported descriptive summaries but are limited in drawing firm conclusions. Some course facilitators are employed elsewhere in the NHS and have incorporated RC activity into their existing roles. Many RCs use significant numbers of unpaid or voluntary staff in core roles. These RCs have an annual budget approximately half of other RCs: £105,000 versus £206,000. It is important to ensure that peer trainers are not exploited, and that RCs are appropriately funded in order to do so. An Australian evaluation incorporating these in-kind costs found that this increased costs per student per course by Australian $50 [18]. The relative cost-effectiveness of facilitating a RC course versus seeing clients for one-to-one sessions within their main NHS role has not been investigated.

## Strengths and limitations

This study has several strengths. The high response rate (72%) means that this survey provides a relatively robust overview of the state of RCs in England in 2021. It is the first national survey to explore commissioning arrangements, workforce and costs. People with lived experience were involved in developing the survey content, both as research team members and through the involvement of the RECOLLECT LEAP who helped shape the questions. A further strength involves the use of a data-driven approach to identify a RC typology.

Several limitations apply. Although CHERRIES reporting guidelines were followed in designing and reporting this survey [22], the use of adaptive questioning (e.g. survey Q60) meant that the number of items and pages could not be reported. The survey took place during the COVID-19 pandemic, when national social distancing restrictions were in place and many RCs were changing to online delivery. This may account for the relatively lower RECOLLECT Fidelity Measure score on the Social Connectedness item. Furthermore, reliance on the RC manager to self-report scores may have resulted in desirability bias, although the spread of RECOLLECT Fidelity Measure scores with no strong ceiling effects does not support this concern. Lastly, we do not have data on non-completing colleges, so we are unable to identify any differences between completers and non-completers.

## Clinical implications

Overall, RCs provide services to nearly 50,000 students in England, at an annual cost of over £20 m. Both their reach and costs are in advance of the formal evidence base and occurs in the absence of any central policy guidance or clinical guidelines support. Further understanding of drivers to their rollout should be identified. The knowledge gaps identified in this survey are being addressed in the RECOLLECT Programme (researchintorecovery.com/recollect) [29]. However, as this work is UK specific and there has been a rollout of RCs in many other countries, further work should be undertaken to establish any cultural and continental variation in organisational and student characteristics, fidelity and running costs internationally to help inform local specific commissioning arrangements.

## Supplementary Information

Below is the link to the electronic supplementary material.

Supplementary file1 (DOCX 274 KB)

## Data Availability

The data that support the findings of this study are available on request from the corresponding author.
