# Supplementary material for: Evidence-based Recovery Colleges: developing a typology based on organisational characteristics, fidelity and funding
Source: Soc Psychiatry Psychiatr Epidemiol. 2023 Mar 11;59(5):759–68. doi: 10.1007/s00127-023-02452-w (PMC10007645; doi:10.1007/s00127-023-02452-w)
Supplement: Supplementary file 1 — Supplementary file1 (DOCX 274 KB) [file 127_2023_2452_MOESM1_ESM.docx]

**Supplementary Materials**

1. Finalised RECOLLECT national survey
2. Reasons for exclusion of organisations from survey
3. Characteristics of students using Recovery Colleges (n=63)
4. Relationship between fidelity score and categorical components (n=63)
5. Cluster analysis results
6. Economic evaluation

**Supplementary material 1**

**Finalised RECOLLECT national survey**

RECOLLECT national survey

**SCREENING QUESTIONS**

Thank you for your interest in this survey about Recovery Colleges in England. We use the term ‘Recovery College’ in this survey but we do recognise that your service may be called something different. Before we ask you to complete the survey, we have four questions about your service to make sure it is eligible to take part in this survey. Please note we’re focused here just on whether your college is in scope for our survey, and we do recognise that your answers won’t be providing a full or complete description e.g. of other goals of your college.

1. **Is your Recovery College based in England?**

- Yes (1)
- No (2)

**2) Is the main focus of your Recovery College on supporting personal recovery?**
[Personal recovery is defined as ‘living as well as possible’ as opposed to e.g. a reduction in symptoms of illness]

- Yes (1)
- No (2)

**3) Does your Recovery College aspire to use co-production at all levels?** 
[Co-production is defined as people with lived experience (e.g. Peer Trainers and students) who work with professionals and subject experts to design and deliver all aspects of the Recovery College. This includes collaborative decision-making about the prospectus, courses, college policies, staff recruitment, advertising, etc., as well as the co-design and co-delivery of all courses by a Peer Trainer and other subject-expert’]

- Yes (1)
- No (2)

**4) Does your Recovery College aspire to use an adult learning approach?**
[Adult learning is defined as an approach whereby students and trainers collaborate and learn from each other by sharing experiences, knowledge, and skills. Students have responsibility for their learning and learn through interactive and reflective exercises. Students gain self-awareness, understanding of their difficulties and practical, relevant self-management skills. Students choose courses which best suit their needs]

- Yes (1)
- No (2)

[If no to any screening questions]

Thank you for expressing an interest in our survey. Unfortunately, your service does not meet our eligibility criteria. If you want to find more out about our work, please visit: <https://www.researchintorecovery.com/research/recollect/>

**PARTICIPANT INFORMATION SHEET (PIS)**

**Study title:**

Recovery Colleges Characterisation and Testing (RECOLLECT): Understanding the development and running of Recovery Colleges in England

**Invitation and brief summary:**

You are invited to take part in RECOLLECT. Before deciding whether to participate it is important to understand the rationale for RECOLLECT and what taking part will involve. Please take the time to read the following information carefully and discuss it with others if you wish. Ask us if there is anything that is not clear or if you would like more information.

**Explanation: purpose and background to the research invitation**

RECOLLECT is a five-year programme of work to better understand Recovery Colleges in England. Over the last decade, Recovery Colleges have rapidly expanded. We first want to build up a national picture of what Recovery Colleges look like in England and how they operate. Later on, we will explore the evidence base around how Recovery Colleges impact outcomes for those that use them.

We are inviting Recovery College Managers and other individuals in a leadership position to take part in RECOLLECT. This invitation is open to all Recovery Colleges in England.

**What would taking part involve?**

As this is a five-year programme of work, there are different ways your Recovery College can be involved. This information sheet is for a survey and optional follow up interview to better understand how Recovery Colleges run and function.

The survey will take about 30 minutes and can be completed online at a time to suit you. It will be sent to your work email address. The survey is hosted by Qualtrics. You will not need to send the survey back to us as survey responses are saved as you progress, however, when you reach the end of the survey, you will reach a page telling you that you have completed the survey. If you are unable to access Qualtrics, we may be able to send you an Excel file to complete to your work email. This would involve you sending the file back when you have finished, to: RECOLLECT@kcl.ac.uk.

As part of this survey, you will be asked questions about your Recovery College, including how it is set up, how it rates on different aspects of Recovery College fidelity, such as co-production, as well as some information about running costs. For the purposes of the project, we will ask for personal information, such as your name and work email address, and ask for consent to keep this so that we know who completed the survey and who to contact about further RECOLLECT research. This will only be available to the RECOLLECT team.

For those that complete the survey, the RECOLLECT team may also contact you later and ask you to take part in an interview. This will be sent to you work address. The aim of this is to explore how and why Recovery Colleges were set-up, what they were like before the pandemic and how it affected them, and what changes are planned for the future. We are aiming to interview 50 individuals in leadership positions in Recovery Colleges. We will ask for consent for this separately if you are interested in taking part and you should sent consent back to us from your work address. These will take place over videocall (Microsoft Teams) and will last approximately 60 minutes. We will audio record the interview so that an approved third-party transcription service can produce a transcript of your responses. This will be analysed by the RECOLLECT team alongside other transcripts so that we can find common themes. Like the survey, we will ask for personal information, such as your name and work email address, so that we know who took part and who to contact about further participation in RECOLLECT. This will only be available to the RECOLLECT team.

Participation in either the survey or interview are completely voluntary. You should only take part if you want to. Choosing not to complete the survey will mean you are unable to take part in an interview or subsequent parts of RECOLLECT as we need this information to sample Recovery Colleges for future work. Other than not being able to take part in any subsequent part of RECOLLECT, you will not be disadvantaged in any other way.

Once you have read the information sheet, please contact us if you have any questions that will help you make a decision about taking part. If you decide to take part in the survey, you will need to consent to your participation and that you are happy for us to contact you about other parts of RECOLLECT, such as an interview (which you are in under no obligation to take part in). If you do decide to take part in an interview, we will ask for your consent to be interviewed separately.

**What are the possible benefits of taking part?**

There will be limited, if any immediate / direct benefit in taking part. However, the findings may have many possible direct / indirect benefits for Recovery College staff / students in the future, such as helping to inform future service provision.

**What are the possible disadvantages and risks of taking part?**

There are no known risks to taking part. Questions in both the survey and interviews are not designed to elicit any emotional response and you are not obliged to take part in other parts of RECOLLECT if you do not want to.

**What if something goes wrong?**

If this project has harmed you in any way or if you wish to make a complaint about the conduct of the project you can contact Nottinghamshire Healthcare NHS Foundation Trust using the details below for further advice and information:

Email: research@nottshc.nhs.uk

Phone: 0115 969 1300

**What will happen if I don’t want to carry on with the study?**

You are free to withdraw your consent to allow us to process any data related to your survey or interview without having to give a reason. If you do change your mind after taking part, you can withdraw any data your provided as part of the survey or the interview. Withdrawing any data that you submitted as part of your response to the survey will mean that you will be unable to take part in other studies in RECOLLECT. However, you may withdraw any data you provided as part of the interview process and still be involved in future work with us.

If you want to withdraw any of your data connected to the survey, please let us know by 1^st^ October 2021. If you want to withdraw any of your data connected to the interview, please let us know by 28^th^ December 2021. After these points, withdrawal of your interview and survey data will not be possible as findings will have been included in analysis and written up in outputs and publications.

**How will my information be kept confidential?**

I understand that my personal information and any information I share during the interview will be held in confidence and not shared with a third party, except the approved transcription service (for interviews only), unless:

1) I share information which suggests that myself or someone else is at risk of harm 2) The Trust are obliged by law to share the information or are required to share the information in response to a court order

3) There is an overriding public interest which requires the Trust to share the information.

During the project, data will be stored on a secure network within the Institute of Psychiatry, Psychology and Neuroscience at Kings College London and may be used for future research when anonymised. Only members of the RECOLLECT team will have access to the full dataset. For interviews, we will share recordings with a third-party approved transcription service to listen to your recording and produce a transcript. No information that could identify you or your Recovery College will be used in any publications or outputs – this means you will both be anonymous. As part of the consent process, we will retain your contact details to make you aware of future participation in RECOLLECT and provide you with findings as the programme progresses. However, you can opt out of further contact at any point.

We will keep identifiable survey data about you (such as your name/email) as well as identifiable information about the Recovery College (survey responses), until the end of the project as we may wish to approach you about taking part in subsequent RECOLLECT work. After this point, the data from this survey will be anonymised. For interview data, transcripts will be anonymised after transcription.

Once the project has ended, these datasets will be held at Nottinghamshire Healthcare NHS Foundation Trust for another 15 years. Researchers from outside of the RECOLLECT team may request access to anonymised data by contacting: research@nottshc.nhs.uk

**What will happen to the results of this study?**

The anonymised results of the project will be summarised in outputs such as project reports, publications and conferences. Findings will be distributed through Recovery College networks (such as ImROC), and appear on the website

www.researchintorecovery.com/research/recollect/publications/

**Who is organising and funding this study?**

The study is being organised by researchers in Kings College London and the University of Nottingham. This project is being funded by the NIHR (Programme Grants for Applied Research, Recovery Colleges Characterisation and Testing (RECOLLECT) 2, NIHR200605).

**Who has reviewed this study?**

The research was reviewed by the sponsor (Nottinghamshire Healthcare NHS Foundation Trust) who confirmed approval from a research ethics committee was not needed as this is a study of usual practice. Further information and contact details If you have any questions or require more information about this project, please contact:

Dr Daniel Hayes

Research Fellow,

RECOLLECT Health Services and Population Research Department P028, David Goldberg Centre, King’s College London Institute of Psychiatry, Psychology and Neuroscience, De Crespigny Park, London SE5 8AF.

Email: RECOLLECT@kcl.ac.uk.

Tel. 07385 466427

**Data Protection**

Your data will be processed in accordance with the UK General Data Protection Regulation (GDPR) and the Data Protection Act 2018. If you would like more information about how your data will be processed in accordance with UK GDPR please visit the links below.

Privacy notice: https://www.nottinghamshirehealthcare.nhs.uk/download.cfm?ver=13335

Further information on processing your personal data: https://www.nottinghamshirehealthcare.nhs.uk/your-information

The sponsor and data controller for this project is Nottinghamshire Healthcare NHS Foundation Trust. King’s College London (KCL) is the Data Processor. Your personal data will only be processed for the purpose of the research outlined within this information sheet. The legal basis for processing your personal data for research purposes under UK GDPR is Article 6: 1I ‘task in the public interest’.

You have the right to access and withdraw information held about you. Your right of access can be exercised in accordance with the UK General Data Protection Regulation. You also have other rights including rights of correction, erasure, objection, and data portability.

Questions, comments and requests about your personal data can be sent to Information Assurance Department at Nottinghamshire Healthcare NHS Foundation Trust[: dpoenquiries@nottshc.nhs](mailto::%20%20dpoenquiries@nottshc.nhs).uk.

**CONSENT FORM**

**Title of Project:**

Recovery Colleges Characterisation and Testing (RECOLLECT): Understanding the development and running of Recovery Colleges in England.

1. I confirm that I have read the participant information sheet dated 14/07/21 (version 1.0) for the above study. I have had the opportunity to consider the information, ask questions and have had these answered satisfactorily.

- Yes

2. I understand that my participation in the survey is voluntary.

- Yes

3. I understand that I can withdraw any information shared as part of the survey, without having to give any reason, up unti^l^ 1st October 2021. After this point, it will not be possible to remove survey data, as it will have been used in analysis and publications/reports.

- Yes

4. I consent to the processing of any information shared as part of the survey for the purposes explained to me in the participant information sheet. I understand that such information will be handled under the terms of UK data protection law, including the UK General Data Protection Regulation (UK GDPR) and the Data Protection Act 2018.

- Yes

5. I understand that it will not be possible to identify me in any project publications or reports as any included data will be anonymised

- Yes

6. I am aware links to project reports or publications, where non-identifiable (anonymised) data may appear, will be available on RECOLLECT website www.researchintorecovery.com/research/RECOLLECT/publications/

- Yes

7. I understand that any information shared as part of the survey may be subject to review by responsible individuals from the Sponsor for monitoring and audit purposes.

- Yes

8. I understand that my any information shared as part of the survey will be held in confidence and not shared with a third party, unless: a) I share information which suggests that myself or someone else is at risk of harm, b) The Trust are obliged by law to share the information or are required to share the information in response to a court order, c) There is an overriding public interest which requires the Trust to share the information

- Yes

9. I agree that the RECOLLECT team may use my anonymised survey data for future research.

- Yes

10. I am aware that the RECOLLECT team will keep identifiable information about me (e.g your name/email) and your Recovery College (e.g. survey responses) until the end of the project. This is so we may invite you to participate in further RECOLLECT related studies. This identifiable information will be stored on a secure network drive at Kings College London during the project, to which only the RECOLLECT team have access. Data will be anonymised at the end of the project.

- Yes

11. I understand that at the end of the project, anonymised data will be kept for 15 years. Other researchers may request access to this anonymised data via agreement with Nottinghamshire Healthcare NHS Foundation Trus[t (research@nottshc.nhs](mailto:t%20(research@nottshc.nhs).uk)

- Yes

12. I consent to the RECOLLECT team contacting me about participation in future RECOLLECT research but that taking part in this survey does not mean I am obliged to take part if approached and that I can opt out of further contact at any point

- Yes

13. I agree to take part in the above study.

- Yes

**SURVEY**

Thank you for giving consent to take part in this survey. We will now ask you questions about your Recovery College (though we know that not all services use this term!). Towards the end of the survey, there are some questions on running costs, so you may need any budgets you have. Please complete all parts, even if some responses are just your best estimate.   We worked with diverse Recovery College stakeholders to create and pilot this survey, but despite our efforts we recognise that response options for some questions may not fully fit your college. We’re very interested in understanding the full range of innovation taking place in Recovery Colleges, so if the available responses don’t quite fit your college, please choose the response which is as close as possible, and make a note of the question. At the end of the survey there will be a chance for you to respond further to these questions.

**Section A) Describing organisational characteristics, curricula and student populations**

**A1) About the college**

These questions are about how your Recovery College runs. Please answer in relation to how the currently runs

**Q1) What is the name of your Recovery College?**

**Q2) What is your name?**

**Q3) What is your role at your Recovery College (e.g. Recovery College Manager)?**

**Q4) What is your email address?**

**Q5) How long has your Recovery College been running (in years)?**

**Q6) What is the annual budget for your Recovery College (approximate if not known)?**

**Q7) Which of these most closely matches the location of your Recovery College?**

- Urban
- Suburban
- Rural
- Mixed

**Q8) Do you have a main physical base (i.e. says Recovery College on the door, with administration, classrooms and library)? [Remember, please ignore any temporary pandemic-related changes to online course delivery)]**

- Yes
- No - we meet in community venues or mixed use venues
- No - we are a virtual college operating only online

**Q9) Besides supporting personal recovery, which of these is the MORE important goal of your Recovery College?**

- To reduce stigma and discrimination in society
- To positively impact on mental health services
- Both are equally important

**Q10) How many courses do you run per year? (Count every time each course is run, e.g. if you run the same course three times, count this as three courses)**

**Q11) How many different courses do you provide? (Count the same course once even if it runs several times)**

**Q12) How many courses does each student typically attend over one year?**

**Q13) Does your recovery college use goal-oriented personal plans (Individual Learning Plans)?**

- Yes
- No
- Unknown/do not know

**Q14) Please upload a copy of your current curriculum**

**A2) Students**

*These questions are about your current student population
**Q15) How many individuals do you expect to register as students in a year in total?  (i.e. count each person once, even if registered for more than one course or across more than one term)**

**Q16 - 22) Who is your Recovery College for?** **Tick all that apply**.

- People with mental health issues who are using no services or only primary care or voluntary sector mental health services
- People with mental health issues who are using secondary mental health services
- People with mental health issues who are using specialist mental health services
- Informal carers (e.g. family, friends) of people with mental health issues
- Mental health worker (e.g. Nurse, Psychologist, Psychiatrist, Counsellor)
- Other staff working in mental health services (e.g. Receptionist, Administrator)
- General public who may have no connection with the mental health system

**Q22-31) Are there particular groups for whom your Recovery College (or part of it, e.g. a specific college campus or spoke) caters? Tick all that apply**.

- Black and Minority Ethnic people
- Children and young people (up to 25)
- Grenfell survivors
- Patients who are in forensic and secure services
- People who are homeless
- People who are unemployed
- People with substance misuse difficulties
- People who are veterans
- Other (e.g. LGBTQIA+)
- We do not cater for distinct groups

**Q32-34) Questions about family and friend caregivers**

- Do you have a designated carers' lead (someone who has a dedicated role to support informal family/friend carers) at your Recovery College?
- Do you routinely monitor whether students are carers?
- In the past two years have you run a course specifically for carers or caring for someone with mental health issues?

**Q35) What is the mean age (years) of the students who attend your Recovery College (if not known, please estimate)**

**Q36-40) Estimate the proportion (% totaling to 100) of students who identify as:**

Asian or Asian British : _______

Black or Black British : _______

Mixed or Mixed British : _______

White or White British : _______

Other : _______

Total : ________

**Q41-44)**Estimate the proportion (% totalling to 100) of students who identify as:

Male : _______

Female : _______

Non-binary / third gender / other : _______

Prefer not to say : _______

Total : ________

**A3) Governance and leadership**

These questions are about the wider organisational context of your Recovery College

**Q45) What is the main organisational affiliation for your Recovery College (e.g. whose buildings you operate from or where data and records is stored (choose one, or more than one if an equal partnership))?**

- Statutory health service, e.g. NHS Trust
- Other health provider, e.g. private healthcare provider
- Local authority, e.g. council
- Education provider, e.g. University or college
- Non-governmental organisation (NGO), Charity, Voluntary o^r^ 3rd Sector
- We are independent
- Other

**Q46) Does your core Recovery College leadership team include people with lived experience of mental health issues?**

- Yes
- No
- Unknown/do not know

**Q47) During coproduction, what groups are most commonly involved? (tick one)**

- Lived experience + health or social care professional
- Lived experience + community topic expert
- Lived experience only
- Other

**Section B) RECOLLECT Fidelity Measure**

Please complete this measure for your main Recovery College, even if you are involved in or managing more than one. Complete it for your Recovery College as it is right now (i.e. including any changes you have had to make due to the pandemic). Make a note of any scores which have changed due to the pandemic as we will ask you about this at the end of this section.

We now list seven dimensions of a Recovery College. Each dimension has three statements describe varying levels of development, from early stage to active engagement to active success. For each dimension, choose the statement which best matches your main Recovery College as it is right now , even if you may have phrased things a little differently. At the end of the survey, there will be the opportunity to tell us more if you found any items difficult to rate.

**Q48) Dimension 1: Valuing equality**

The contributions and assets of students, trainers (peers, clinicians, external) and other staff are equally valued. No one is judged or treated differently because of their background or mental health difficulties

- We recognise that staff and students may take time to develop partnership-based working relationships. Whilst being supportive of staff and students, we only deal with issues of discrimination and power differences when they arise.
- We do not actively ensure that all relationships in the college demonstrate equal sharing of opportunities, training, etc. However, we do ensure that the college is welcoming to all staff and students, and have some structures in place (e.g. open days, training, supervision) to encourage equality and to challenge stigma and discrimination.
- We actively promote a non-judgemental and welcoming culture. Activities are undertaken to ensure that issues of power are always considered within the college (e.g. equal access to training and resources, diversity in promotional materials, analysing equal opportunity data).

**Q49) Dimension 2: Learning**

Recovery Colleges follow an adult education approach whereby students and trainers collaborate and learn from each other by sharing experiences, knowledge and skills. Students have responsibility for their learning and learn through interactive and reflective exercises. Students gain self-awareness, understanding of their difficulties and practical, relevant self-management skills. Students choose courses which best suit their needs

- We cannot provide evidence of the college’s model(s) of adult learning. We can identify a large number of barriers to progress, such as the influence of a strong clinical or psychoeducational model, or limited resources for Peer Trainer training. Trainers are skilled in delivering education and encouraging shared learning.
- We can articulate the college’s model(s) of adult learning. Some processes are in place to ensure that trainers follow educational principles (e.g. lesson plans, educational language) and that courses involve co-learning. However, some barriers prevent the full and effective implementation of these model(s), e.g. time pressures to launch/recruit to new courses, or barriers to trainer recruitment and training.
- We can demonstrate the college’s full commitment to principles of adult learning. These are evident in the college’s prospectus, curriculum and course materials. All trainers (including clinical trainers) can describe the model(s) of adult learning used in the college, and are offered ongoing formal or accredited training in adult learning

**Q50) Dimension 3: Tailored to the student**
Recovery Colleges don’t offer a one size-fits-all experience. Students’ individual needs are actively enquired about and accommodated during courses (e.g. personalised handouts, translated text, materials adapted for learning difficulties). Their needs outside the course are also accommodated (e.g. buddy service, transport help, individual learning plans).

- We are not able to demonstrate the ways in which the college provides an individualised experience for students. Trainers are not actively supported or trained to take account of and accommodate student differences during classes.
- We can demonstrate some ways in which individual needs of students are addressed, but recognise that there are still unmet needs, e.g. students with learning difficulties or nonfluent English speakers.
- We are able to demonstrate many ways in which students’ individual needs are addressed both during and outside courses. Trainers are made aware of students' needs in advance and provided with guidance on how to adapt the content/delivery of courses.

**Q51) Dimension 4: Co-production of the Recovery College**

People with lived experience (Peer Trainers and students) are brought together with professionals and subject experts to design and deliver all aspects of the Recovery College. This includes collaborative decision making about the prospectus, courses, college policies, staff recruitment, advertising, etc., as well as the co-design and co-delivery of all courses by a Peer Trainer and other subject-expert

- We routinely involve students and staff in decision-making about the design and running of the Recovery College. Most of our success in co-production has been at the level of course co-delivery. We recognise that there are currently some significant barriers to co-production throughout the college, including those of culture, management hierarchies and time
- As well as consistent co-delivery of courses, we involve staff and students in most discussions about the design and running of the Recovery College (e.g. through student steering groups or student reps), but managers make many of the decisions.
- We can demonstrate a culture of co-production and its consistent use across the college. The voices of trainers and students are equally heard during decision-making across all levels of the college, including co-delivery, curriculum development, management and design of the physical environment.

**Q52) Dimension 5: Social connectedness**

Both the culture and the physical environment of the college provide students with opportunities to develop connections with others. The learning space is relaxed, e.g. nonclinical chair layout, access to drinks facilities, shared spaces for socialising. Trainers recognise and cater for students' social needs, e.g. organising exercises and breaks for chatting, sharing experiences and
 developing friendships

- Students' social experience is low on the Recovery College’s agenda when deciding on course structure and the physical environment. There are no specific processes for students to get to know one another. Course venues rarely have facilities or spaces outside the classroom where students can relax or socialise.
- We ensure that the Recovery College is a welcoming environment for students. Trainers are encouraged to provide opportunities for socialising during courses where possible, but this is not central to their role. A few of our course spaces have facilities outside the classroom where students can relax, but there are a number of practical or financial barriers to this.
- The Recovery College recognises the role that student integration and connectedness plays in learning and recovery. The college provides a range of facilities for socialising (e.g. café, seating areas, informal and spacious course venues). Trainers are supported to integrate opportunities for students to form closer bonds with each other into the structure of courses

**Q53) Dimension 6: Community focus**

Recovery Colleges engage with community organisations (e.g. mental health charities, artistic/sporting groups) and Further Education colleges to co-produce relevant courses. The college provides students with information, handouts and events which support students' pathways into valued activities, roles, relationships and support in the community.

- We have limited involvement with, or presence in, community organisations. Community organisations are not involved in college meetings or events, or do not routinely work with the college to co-produce courses or facilitate opportunities for staff/students.
- We ensure that the college undertakes some activities to build awareness of its community services and relationships with community organisations. Some college courses are coproduced with community organisations and students are signposted to relevant community organisations for support.
- We work with a range of community organisations to co-produce college courses and facilitate pathways for students. We can demonstrate activities to build awareness of, and relationships with, the community. We can demonstrate that joint-working with community organisations has led to changes in the college

**Q54) Dimension 7: Commitment to recovery**

Recovery College workers talk with conviction and enthusiasm about the service and are dedicated to students' recovery. There is a positive energy in the college and its activities, based on shared values about the recovery principles on which the college is based.

- Our organisational policies and procedures ensure the Recovery College runs smoothly, but there are barriers (e.g. culture, organisational structures) to personal investment by workers in promoting recovery principles (dimensions 1 to 6 above) throughout the college. There is still significant effort needed to establish the college as something ‘different’ and ‘meaningful’.
- We actively motivate each other to promote recovery principles. We have a shared commitment to constantly improve the recovery focus of the college but recognise some barriers to progress (e.g. cultural, financial).
- We actively promote recovery principles in the college, and collectively lead with enthusiasm and an expressed belief in the college’s students and staff. College activities demonstrate recovery principles in practice, e.g. graduation ceremonies, students becoming trainers.

We now ask you about five components which can differ between Recovery Colleges. Please pick the type that most closely resembles your college as it is right now.

**Q55) Component 1: Available to all**

- Type 1: The Recovery College is available to all. The Recovery College is accessible to any adult (16+ or 18+), including staff and carers, regardless of their use of local services of any kind. Any restrictions are minimal, e.g. living locally, being registered with a GP.
- Type 2: The Recovery College is limited to specific groups. The Recovery College is open to adults (16+ or 18+) who are current or previous users of local secondary care mental health services. There may be local additions to this eligibility e.g. health/social care/community organisation staff, or family and carers. Being ‘inclusive’ relates to the ways in which the Recovery College does not discriminate or create access barriers for people with, for example, certain diagnoses, learning difficulties or physical health/mobility needs

**Q56) Component 2:** **Location**

- Type 1: The Recovery College is based in a community location that is not shared with health, social care or other statutory services. The Recovery College is deliberately located within communities or neighbourhoods, not in NHS or social care buildings.
- Type 2: The Recovery College is based in a location which is shared with health, social care or other statutory services. The Recovery College is located within or near (e.g. adjoining building) to local NHS or social care services

**Q57) Component 3: Distinctiveness of course content**

- Type 1: Any topic can be offered as a course, irrespective of whether it is available in mainstream adult education settings. The curriculum includes courses on topics which are also available in local mainstream colleges. Example courses might include gardening, arts, Maths, English, budgeting, understanding benefits, physical health care, job-seeking, home maintenance and a range of leisure/recreation activities.
- Type 2: Only topics not available in mainstream adult education settings are offered. The curriculum never includes courses on topics which are available in local mainstream colleges. Some courses are offered with a specific recovery-related focus, e.g. gardening for wellbeing, arts for recovery

**Q58) Component 4:** **Strengths-based**

- Type 1 A focus on strengths (not problems) is implicit in the college. The learning opportunities offered by the Recovery College implicitly builds on the experiences, strengths, assets and resources of students. The language of being ‘strengths-based’ is not often used.
- Type 2 A focus on strengths (not problems) is explicit in the college, in addition to dimensions 1-7 above. The learning opportunities offered by the Recovery College explicitly build on the experiences, strengths, assets and resources of students. The language of being ‘strengths-based’ is routinely used by staff and students, and features in course materials and other aspects of the Recovery College.

**Q59) Component 5:** **Progressive**

- Type 1 There is a focus on ‘being’ and ‘belonging’, not on goal-setting. The focus of the Recovery College is on supporting individual students' learning needs, safety and belonging, identity development, personal meaning-making and reflection. The college does not require behavioural goal-setting. Students can learn in whatever direction they want to – and for some students that might not be about moving forwards.
- Type 2 There is a focus on ‘becoming’ and a strong emphasis on goal-setting and change. The focus of the Recovery College is on processes which provide pathways of opportunity for students and which support them to move on with their lives. This might include the use of goal-oriented personal plans (Individual Learning Plans) and planning and reviewing goal-oriented activities

**Q60) Do you think your Recovery College would have scored differently on any of these domains prior to the pandemic?**

- Yes
- No

*Skip To: Q73 How is your College funded if Q60? = No*

**Q61-72) Which do you think you would have scored differently on (tick all that apply)?**

- Dimension 1: Valuing equality
- Dimension 2: Learning
- Dimension 3: Tailored to the student
- Dimension 4: Co-production of the Recovery College
- Dimension 5: Social connectedness
- Dimension 6: Community focus
- Dimension 7: Commitment to recovery
- Component 1: Available to all
- Component 2: Location
- Component 3: Distinctiveness of course content
- Component 4: Strengths-based
- Component 5: Progressive

**Section C) Organisational costs**

We are trying to learn more about how much it costs to run a recovery college and how much of that money is spent on different types of resources. So for this section, if you are in the first 6 months of your financial year then please answer these questions about the current financial year. If you are in the last 6 months of your financial year then please answer these questions about the next financial year. We understand that some recovery colleges are integrated within other services, but in your responses please give information just about the recovery college budget as far as possible.

**Q73-78) How is your college funded (indicate the percentage of funding received from all applicable funding sources below)? If you don’t know exactly, please report approximate percentage of funding from any applicable sources. Please ensure the total equals 100**

Clinical commissioning group (CCG), integrated care system (ICS), or equivalent :

NHS Trust:

Charitable fund, grants, and/or donations:

Self-funded (e.g. charges for courses; fund-raising activities by staff/students):

Independent provider services:

Other:

Total:

**Q79) Are there additional relevant details about these funders you wish to tell us?**

**Q80) How much do you pay per year to use any indoor/outdoor premises (e.g. rent)? If you do not pay anything, please write ‘0' in the box.**

**Q81) What is your annual budget for technology (e.g. laptops/e-learning software) and stationery costs, which are not included in premises costs? If you do not pay anything, please write ‘0' in the box**

**Q82) What is your annual budget for central/core staff salaries? (i.e. those contracted for regular employment by the recovery college)?**

**Q83-86) Who are the staff in the central/core team?**
Please specify the **whole time equivalent (WTE)** (i.e. full working week - if two people each work 2.5 days per week this is equivalent to 1 whole time equivalent). Please indicate the NHS Agenda for change band that reflects the majority of staff in the respective roles – for non-NHS staff, indicate the equivalent NHS Agenda for change band based on their salary. Please write 0 if not relevant and not applicable (after Band 9) in the salary section

| Role | Band/Salary |
| --- | --- |
| Manager(–) - Number of FTE (1) |  |
| Administrator(–) - Number of FTE (2) |  |
| Peer trainer(s)/learning advisor(–) - Number of FTE (3) |  |
| Other trainer(–) - Number of FTE (4) |  |

**Q87) Are there any other paid core roles at your Recovery College?**

- Yes
- No

*Skip To: Q116 if Are there any other paid core roles at your Recovery College? = No*

**Q88-115) Please list any other paid core roles. Please do not report any sessional/occasional staff here, we will ask about these roles further down. Please write 0 if not relevant**

Role:

Number of paid individuals:

Typical number of hours per week:

NHS Agenda for Change pay band (or equivalent)

**Q116) Are there any unpaid core roles at your Recovery College?**

- Yes
- No

*Skip To: Q138 If Q116) Are there any unpaid core roles at your Recovery College? = No*

**Q117-137) Please list any unpaid core roles. Please do not report any sessional/occasional staff here, we will ask about these roles in the next question. Please write 0 if not relevant**

Role

Number of individuals who volunteer

Typical number of hours per week

**Q138-162) Who else does work in the Recovery College and typically how many hours do they work there over a year (e.g. a Consultant Psychologist teaching a course once a year)? Please tell us about sessional/occasional staff not included in the previous questions here.  If you do not have these roles, please put ‘0’ in each corresponding box.**

Role (e.g. Nurse)

Does anyone in this role work at the Recovery College?

Does the Recovery College pay directly for this role?

Total number of hours per year

**Q163) What is the Recovery College annual budget for staff training? If you do not pay anything, please put ‘0’ in the box**

**Q164) Have you paid for or submitted any business cases for important new staff/facilities/equipment to support the delivery of the recovery college that you haven’t listed above?**

- Yes
- No

**Q165) If yes, please provide more information**

**Q166) Has the COVID-19 pandemic had an impact on your budget/spending**

- Yes
- No

**Q167) If yes, please provide more information**

**167) If you have answered 'other' to any question in the survey or would like to tell us more about any of your responses, please do so here:**

**Q168) To make sure we have contacted all Recovery Colleges in England, please give any names and contact details of other Recovery Colleges in your County / City / Borough:**

**Supplementary material 2**

**Reasons for exclusion of organisations from survey**

| **Reasons for exclusion of identified organisations** | **Number of excluded organisations** |
| --- | --- |
| Non-contactable and local/expert contacts believe it does not exist | 20 |
| Duplicate name for already-included Recovery College | 6 |
| Satellite site of an included Recovery College | 5 |
| Existing but not currently running | 5 |
| Previously open but now closed | 4 |
| Not a Recovery College (e.g. day unit) | 4 |
| Merged with another Recovery College | 1 |
| Just opened and unable to complete the survey | 1 |
| **Total** | **46** |

**Supplementary material 3**

**Characteristics of students using Recovery Colleges (n=63)**

| **Student characteristic** | **Mean±SD, N (%)** |
| --- | --- |
| Number of students per year (median, IQR)  Range | 300 (125-575)  50 to 4,919 |
| Age of students (years) | 40.7±6.9 |
| Gender proportion (52 colleges) |  |
| *Male* | 36.2±16.7 |
| *Female* | 57.5±18.3 |
| *Non-binary/third gender* | 1.9±3.1 |
| *Prefer not to say* | 45±11.8 |
| Ethnicity proportion (56 colleges) |  |
| *Asian/Asian British* | 6.4±6.6 |
| *Black/Black British* | 8.9±10.8 |
| *Mixed/Mixed British* | 5.5±6.0 |
| *White/White British* | 71.5±24.0 |
| *Other* | 7.8±13.2 |
| Who recovery college is for: |  |
| *People with mental health issues who are using no services or only primary care or voluntary sector mental health services* | 48 (76) |
| *People with mental health issues who are using secondary mental health services* | 58 (92) |
| *People with mental health issues who are using specialist mental health services* | 61 (97) |
| *Informal carers (e.g. family, friends) of people with mental health issues* | 58 (92) |
| *Mental health worker (e.g. Nurse)* | 57 (91) |
| *Other staff working in mental health services (e.g, Receptionist)* | 55 (87) |
| *General public who may have no connection with the mental health system* | 41 (65) |
| Does recovery college cater for specific groups? |  |
| *Black and minority ethnic groups* | 17 (27) |
| *Children and young people* | 15 (24) |
| *Forensic* | 17 (27) |
| *Homeless* | 11 (18) |
| *Unemployed* | 18 (29) |
| *Substance abuse* | 19 (30) |
| *Veterans* | 12 (19) |
| *Other (e.g. LGBTQIA+)* | 19 (30) |
| *Do not cater for distinct groups* | 33 (52) |
| Designated carer’s lead? |  |
| *Yes* | 18 (29) |
| Monitoring whether students are carers? |  |
| *Yes* | 42 (67) |
| Carer-specific course in past 2 years? |  |
| *Yes* | 42 (67) |

**Supplementary material 4**

**Relationship between fidelity score and categorical components (n=63)**

| **Item** | **Type** | **Fidelity score**  M±SD | **p value**  Bold = p<0.05 |
| --- | --- | --- | --- |
| Available to all | Type 1 [Anyone] | 11.0±2.4 | 0.102 |
|  | Type 2 [Specific groups] | 9.8±2.9 |  |
| Location | Type 1 [Community] | 11.6±1.9 | **0.003** |
|  | Type 2 [Statutory] | 9.8±2.8 |  |
| Distinctiveness | Type 1 [Mainstream] | 10.6±2.8 | 0.878 |
|  | Type 2 [Not mainstream] | 10.7±2.4 |  |
| Strengths | Type 1 [Implicit] | 9.1±3.0 | **0.012** |
|  | Type 2 [Explicit] | 11.1±2.3 |  |
| Progressive | Type 1 [No goal setting] | 10.3±2.6 | 0.109 |
|  | Type 2 [Goal setting] | 11.4±2.4 |  |

**Supplementary material 5**

**Figure S5.1: Dendrogram to support cluster analysis**


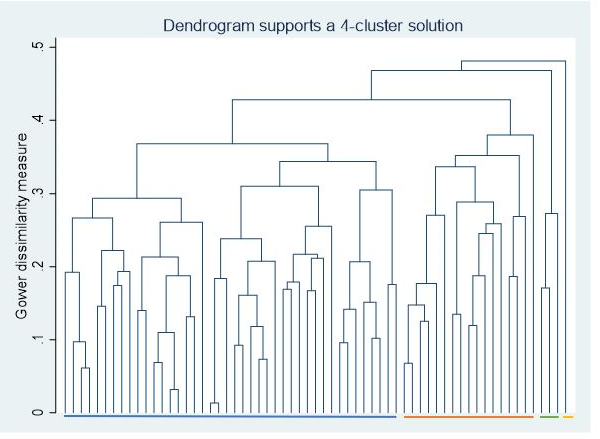


**Table S5.1: Organisational characteristics of Recovery Colleges (n=63) by cluster**

|  | **Sample** | **Cluster 1** | **Cluster 2** | **Cluster 3** | **Cluster 4** |  |
| --- | --- | --- | --- | --- | --- | --- |
|  | M±SD, N(%) | M±SD, N(%) | M±SD, N(%) | M±SD, N(%) | M±SD, N(%) | p value  Bold = p<0.05 |
| Number (%) of colleges | 63 (100) | 42 (67) | 17 (27) | 3 (5) | 1 (2) |  |
| Time in operation (years) | 5.60±2.34 | 5.69±2.28 | 5.18±2.61 | 7.00±2.00 | 4.80±0.00 | 0.624 |
| **Location** |  |  |  |  |  | **0.025** |
| *Urban* | 21 (33) | 12 (29) | 6 (35) | 2 (67) | 1 (100) |  |
| *Suburban* | 5 (8) | 1 (2) | 3 (18) | 1 (33) | 0 (0) |  |
| *Rural* | 2 (3) | 0 (0) | 2 (12) | 0 (0) | 0 (0) |  |
| *Mixed* | 35 (56) | 29 (69) | 6 (35) | 0 (0) | 0 (0) |  |
| Main physical base |  |  |  |  |  | 0.530 |
| *Yes* | 32 (51) | 20 (48) | 10 (59) | 2 (67) | 0 (0) |  |
| *No –community/mixed-use venues* | 30 (48) | 22 (52) | 6 (35) | 1 (33) | 1 (100) |  |
| *No – virtual college online* | 1 (2) | 0 (0) | 1 (6) | 0 (0) | 0 (0) |  |
| More important goal of recovery college |  |  |  |  |  | 0.052 |
| *To reduce stigma and discrimination in society* | 5 (8) | 3 (7) | 1 (6) | 0 (0) | 1 (100) |  |
| *To positively impact on MH services* | 1 (2) | 1 (2) | 0 (0) | 0 (0) | 0 (0) |  |
| *Both are equally important* | 57 (91) | 38 (91) | 16 (94) | 3 (100) | 0 (0) |  |
| Goal-oriented personal plans |  |  |  |  |  | 0.189 |
| *Yes* | 30 (48) | 19 (45) | 10 (59) | 0 (0) | 1 (100) |  |
| *No* | 33 (52) | 23 (55) | 7 (41) | 3 (100) | 0 (0) |  |
| **Main organisational affiliation** |  |  |  |  |  | **<0.001** |
| *Statutory health service, e.g., NHS Trust* | 43 (68) | 39 (93) | 1 (5) | 2 (67) | 1 (100) |  |
| *Other* | 20 (32) | 3 (7) | 16 (94) | 1 (33) | 0 (0) |  |
| Leadership team with lived experience |  |  |  |  |  | 0.227 |
| *Yes* | 59 (92) | 38 (91) | 17 (100) | 2 (67) | 1 (100) |  |
| *No* | 5 (8) | 4 (10) | 0 (0) | 1 (33) | 0 (0) |  |
| Groups involved in coproduction |  |  |  |  |  | 0.407 |
| *Lived experience + health/social care professional* | 45 (71) | 33 (79) | 8 (47) | 3 (100) | 1 (100) |  |
| *Lived experience + community topic expert* | 12 (19) | 7 (17) | 5 (29) | 0 (0) | 0 (0) |  |
| *Lived experience only* | 4 (6) | 1 (2) | 3 (18) | 0 (0) | 0 (0) |  |
| *Other* | 2 (3.2) | 1 (2.4) | 1 (5.9) | 0 (0) | 0 (0) |  |

**Table S5.2: Student characteristics of Recovery Colleges (n=63) by cluster**

|  | **Sample** | **Cluster 1** | **Cluster 2** | **Cluster 3** | **Cluster 4** |  |
| --- | --- | --- | --- | --- | --- | --- |
|  | M±SD,  N (%) | M±SD, N (%) | M±SD, N (%) | M±SD,  N (%) | M±SD,  N (%) | p value  Bold = p<0.05 |
| Number (%) of colleges | 63 (100) | 42 (67) | 17 (27) | 3 (5) | 1 (2) |  |
| Students per year (median, (IQR)) | 300 (125-575) | 375 (180-575) | 300 (155-600) | 60 (50-65) | 100 | 0.625 |
| Range | 50 to 4,919 | 69 to 4,919 | 50 to 1,500 | 50 to 65 |  |  |
| Age of students (years) | 40.7±7.0 | 40.4±7.2 | 41.9±6.5 | 36.7±7.6 | 44.5±0.0 | 0.613 |
| Gender proportion (52 colleges) |  |  |  |  |  |  |
| ***Male*** | 36.2±16.7 | 35.8±10.5 | 37.3±13.1 | 83.3±20.8 | - | **<0.001** |
| ***Female*** | 57.5±18.3 | 60.6±14.3 | 58.5±17.2 | 16.0±19.7 | - | **0.038** |
| *Non-binary/third gender* | 1.9±3.1 | 2.3±3.5 | 1.1±1.8 | 0.7±1.1 | - | 0.579 |
| *Prefer not to say* | 4.5±11.8 | 5.4±13.8 | 3.3±5.7 | 0 | - | 0.811 |
| Ethnicity proportion (56 colleges) |  |  |  |  |  |  |
| *Asian/Asian British* | 6.4±6.6 | 6.8±7.3 | 4.8±3.2 | 8.3±10.4 | - | 0.591 |
| *Black/Black British* | 8.9±10.8 | 9.0±10.7 | 6.4±8.8 | 19.5±18.7 | - | 0.211 |
| *Mixed/Mixed British* | 5.5±6.0 | 5.7±6.5 | 4.3±3.4 | 8.3±10.4 | - | 0.569 |
| *White/White British* | 71.5±24.0 | 72.2±23.4 | 71.6±24.4 | 62.2±37.4 | - | 0.928 |
| *Other* | 7.8±13.3 | 6.2±7.1 | 12.9±22.7 | 1.7±2. 9 | - | 0.452 |
| Who recovery college is for: |  |  |  |  |  |  |
| ***People with mental health issues who are using no services or only primary care or voluntary sector mental health services*** | 48 (76) | 30 (71) | 17 (100) | 0 (0) | 1 (100) | **0.001** |
| ***People with mental health issues who are using secondary mental health services*** | 58 (92) | 40 (95) | 16 (94) | 1 (33) | 1 (100) | **0.002** |
| *People with mental health issues who are using specialist mental health services* | 61 (97) | 40 (95) | 17 (100) | 3 (100) | 1 (100) | 0.793 |
| ***Informal carers (e.g. family, friends) of people with mental health issues*** | 58 (92) | 39 (93) | 17 (100) | 1 (33) | 1 (100) | **0.001** |
| *Mental health worker (e.g. Nurse)* | 57 (91) | 38 (91) | 15 (88) | 3 (100) | 1 (100) | 0.914 |
| *Other staff working in mental health services (e.g, Receptionist)* | 55 (87) | 38 (91) | 14 (82) | 2 (67) | 1 (100) | 0.561 |
| *General public who may have no connection with the mental health system* | 41 (65) | 29 (69) | 11 (65) | 0 (0) | 1 (100) | 0.093 |
| Does your recovery college cater for specific groups? |  |  |  |  |  |  |
| *Black and minority ethnic groups* | 17 (27) | 13 (31) | 2 (12) | 2 (67) | 0 (0) | 0.165 |
| *Children and young people* | 15 (24) | 10 (24) | 3 (18) | 1 (33) | 1 (100) | 0.295 |
| ***Forensic*** | 17 (27) | 10 (24) | 4 (24) | 3 (100) | 0 (0) | **0.032** |
| *Homeless* | 11 (18) | 9 (21) | 2 (12) | 0 (0) | 0 (0) | 0.640 |
| *Unemployed* | 18 (29) | 13 (31) | 3 (18) | 2 (67) | 0 (0) | 0.303 |
| *Substance abuse* | 19 (30) | 14 (33) | 2 (12) | 2 (67) | 1 (100) | 0.067 |
| *Veterans* | 12 (19) | 9 (21) | 2 (12) | 1 (33) | 0 (0) | 0.712 |
| *Grenfell survivors* | 3 (5) | 3 (7) | 0 (0) | 0 (0) | 0 (0) | 0.665 |
| *Other (e.g., LGBTQIA+)* | 16 (25) | 11 (26) | 4 (24) | 1 (33) | 0 (0) | 0.922 |
| *Do not cater for distinct groups* | 33 (52) | 23 (55) | 10 (59) | 0 (0) | 0 (0) | 0.189 |

**Table S5.3: RECOLLECT Fidelity Measure scores (n=63) by cluster**

|  | **Sample** | **Cluster 1** | **Cluster 2** | **Cluster 3** | **Cluster 4** |  |
| --- | --- | --- | --- | --- | --- | --- |
|  | N (%) | N (%) | N (%) | N (%) | N (%) | p value  Bold = p<0.05 |
| Number (%) of colleges | 63 (100) | 42 (67) | 17 (27) | 3 (5) | 1 (2) |  |
| **ORDINAL COMPONENTS** |  |  |  |  |  |  |
| **Equality** |  |  |  |  |  | **<0.001** |
| *High* | 50 (79) | 36 (86) | 13 (77) | 1 (33) | 0 (0) |  |
| *Med* | 12 (19) | 6 (14) | 4 (24) | 2 (67) | 0 (0) |  |
| *Low* | 1 (2) | 0 (0) | 0 (0) | 0 (0) | 1 (100) |  |
| Adult learning |  |  |  |  |  | 0.152 |
| *High* | 30 (48) | 19 (45) | 10 (59) | 0 (0) | 1 (100) |  |
| *Med* | 30 (48) | 21 (50) | 7 (41) | 2 (67) | 0 (0) |  |
| *Low* | 3 (4) | 2 (5) | 0 (0) | 1 (33) | 0 (0) |  |
| Tailored to the student |  |  |  |  |  | 0.711 |
| *High* | 3 (51) | 21 (50) | 9 (47) | 2 (67) | 0 (0) |  |
| *Med* | 31 (49) | 21 (50) | 8 (53) | 1 (33) | 1 (100) |  |
| *Low* | 0 (0) | 0 (0) | 0 (0) | 0 (0) | 0 (0) |  |
| Co-production |  |  |  |  |  | 0.104 |
| *High* | 40 (64) | 28 (67) | 12 (71) | 0 (0) | 0 (0) |  |
| *Med* | 19 (30) | 11 (26) | 5 (29) | 2 (67) | 1 (100) |  |
| *Low* | 4 (6) | 3 (7) | 0 (0) | 1 (33) | 0 (0) |  |
| **Social connectedness** |  |  |  |  |  | **0.047** |
| *High* | 27 (43) | 16 (38) | 11 (65) | 0 (0) | 0 (0) |  |
| *Med* | 27 (43) | 21 (50) | 4 (24) | 2 (67) | 0 (0) |  |
| *Low* | 9 (14) | 5 (12) | 2 (12) | 1 (33) | 1 (100) |  |
| **Community focus** |  |  |  |  |  | **0.004** |
| *High* | 33 (52) | 23 (55) | 10 (59) | 0 (0) | 0 (0) |  |
| *Med* | 23 (37) | 16 (38) | 6 (35) | 2 (67) | 0 (0) |  |
| *Low* | 7 (11) | 3 (7) | 1 (6) | 1 (33) | 1 (33) |  |
| **Commitment to recovery** |  |  |  |  |  | **<0.001** |
| *High* | 44 (69.8) | 31 (73.8) | 13 (76.5) | 0 (0) | 0 (0) |  |
| *Med* | 17 (27.0) | 11 (26.2) | 4 (23.5) | 2 (66.7) | 0 (0) |  |
| *Low* | 2 (3.2) | 0 (0) | 0 (0) | 1 (33.3) | 1 (100.0) |  |
| **Fidelity score** (Median, (IQR))  Range | 11 (9-13)  3 to 14 | 11 (9-12)  6 to 14 | 12 (9-13)  8 to 14 | 6 (3-9)  3 to 9 | 4 | **<0.001** |
| **CATEGORICAL COMPONENTS** |  |  |  |  |  | **0.034** |
| **Available to all** |  |  |  |  |  | **0.034** |
| *Type 1 (Anyone)* | 44 (70) | 29 (69) | 14 (82) | 0 (0) | 1 (100) |  |
| *Type 2 (Specific groups)* | 19 (30) | 13 (31) | 3 (18) | 3 (100) | 0 (0) |  |
| **Location** |  |  |  |  |  | **<0.001** |
| *Type 1 (Community)* | 30 (48) | 13 (31) | 17 (100) | 0 (0) | 0 (0) |  |
| *Type 2 (Statutory)* | 33 (52) | 29 (69) | 0 (0) | 3 (100) | 1 (100) |  |
| Distinctiveness of course content |  |  |  |  |  | 0.097 |
| *Type 1 (Mainstream)* | 27 (43) | 15 (36) | 9 (53) | 3 (100) | 0 (0) |  |
| *Type 2 (Not mainstream)* | 36 (57) | 27 (64) | 8 (47) | 0 (0) | 1 (100) |  |
| **Strengths** |  |  |  |  |  | **0.001** |
| *Type 1 (Implicit)* | 13 (21) | 4 (10) | 6 (35) | 3 (100) | 0 (0) |  |
| *Type 2 (Explicit)* | 50 (80) | 38 (91) | 11 (65) | 0 (0) | 1 (100) |  |
| Progressive |  |  |  |  |  | 0.084 |
| *Type 1 (No goal setting)* | 41 (65) | 30 (71) | 8 (47) | 3 (100) | 0 (0) |  |
| *Type 2 (Goal-setting)* | 22 (35) | 12 (29) | 9 (53) | 0 (0) | 1 (100) |  |

**Supplementary material 6**

**Economic evaluation**

**Budget by college clusters**

The budgetary overview for Clusters 1-3 is reported in Table S6.1.

**Table S6.1: Annual budgets, students and courses per college (n=62) by cluster**

|  | **Cluster 1**  Median (IQR) | **Cluster 2**  Median (IQR) | **Cluster 3**  Values reported |
| --- | --- | --- | --- |
| N | 42 | 17 | 3 |
| Annual budget | n=34  £190,000  (£127,000-338,000) | n=15  £206,000  (£92,000-241,300) | n=1  £150,000 |
| Number of students | n=42  375  (180-575) | n=17  300  (155-600) | 50  60  65 |
| Number of courses (total run per year) | n=41  150  (70-220) | n=17  120  (70-220) | 20  60  118 |
| Number of individual courses | n=42  40  (27-50) | n=17  25  (19-35) | 18  40  72 |
| Number of courses per student | n=31  5  (4-10) | n=11  4  (3-5) | 3  4  36 |
| Cost per student | n=34  £504  (£250-807) | n=15  £618  (£275-875) | n=1  £2,308 |
| Cost per course run | n=34  £1,257  (£682-3,030) | n=15  £1,750  (£649-3,667) | n=1  £2,500 |
| Cost per course designed | n=34  £5,556  (£2,356-8,333) | n=15  £6,667  (£4,333-11,667) | n=1  £3,750 |

Cluster 3 contains 3 colleges, only one of which reported an overall budget therefore not much can be inferred about this cluster. The median annual budget was similar between Cluster 1 and 2. The median number of students, number of courses, and number of courses per student were 25% higher in Cluster 1 than Cluster 2. The number of individual courses offered by the colleges in Cluster 1 was 60% higher than in Cluster 2. The costs per student and per course were higher in Cluster 2 colleges than in Cluster 1.

**Funding source**

There are a variety of patterns of funding reported by Recovery Colleges, summarised in Table S6.2.

**Table S6.2: Funding sources reported by colleges (n=59)**

|  | **Clinical Commissioning Group (CCG)** | **National Health Service (NHS) Trust** | **Charity** | **Self-funded** | **Local authority/ council** |
| --- | --- | --- | --- | --- | --- |
| **COLLEGES WITH ONE FUNDING SOURCE (n=35)** |  |  |  |  |  |
| Number of colleges n (%) | 12 (34) | 15 (43) | 3 (9) | 1 (3) | 4 (11) |
|  | *Median (IQR)*  *Range* | *Median (IQR)*  *Range* | *Individual responses* | *Individual responses* | *Individual responses* |
| Annual budget | £200,000  (£145,000-£241,300)  £90,000-£345,000 | £205,000  (£150,000-£338,000) £15,000-£640,000 | £4,000  £279,500  £555,000 | £15,000 | £130,000  £255,000  £425,000 |
| **COLLEGES WITH MORE THAN ONE FUNDING SOURCE (n=24)** | *n* | *n* | *n* | *n* | *n* |
| Number of colleges with two funders (n=17) | 10 | 13 | 7 | 2 | 2 |
| Number of colleges with three or more funders (n=7) | 4 | 5 | 7 | 1 | 3 |

Of the 59 colleges who reported their funding sources, most had only one funding source (35/59; 59%), followed by two funders (17/59; 29%). Six colleges (10%) reported three funding sources and one reported four funding sources. The median budget in those with one funder was £200,000 (IQR £130,000-300,000). For colleges with two funders, the median budget was £178,581 (IQR £66,000-£335,000). For colleges with three or more funders, the median budget was £150,000 (IQR £45,000-£350,000). Amongst the colleges with a single funder, NHS-funding was by far the most common with either a CCG or NHS Trust being the sole funder for 27 colleges; 77% of those with a single funder. A small number of colleges were solely funded by a local authority/council (n=4) or charity (n=3). For colleges with two funders, the NHS (CCG or Trust) was the most common contributor to funds, followed by charity. For colleges with three or more funders, charity was the most common source.

The funders contributing to colleges with two funding sources are broken down in Table S6.3.

**Table S6.3: Combinations of funders and proportion of funding by source for colleges with two-funders (n=17)**

|  | **CCG**  **and**  **NHS Trust** | **CCG and Charity** | **CCG**  **and**  **Local authority / council** | **NHS Trust and Charity** | **NHS Trust and**  **self-funded** |
| --- | --- | --- | --- | --- | --- |
| Number of colleges with this combination | 6 | 2 | 2 | 5 | 2 |
| Funder: | **CCG** | **NHS Trust** | **Charity** | **Self-funded** | **Local authority/ council** |
| Most frequent proportion of funding from this source | 50% | 50% | 20% | 30% | 10%  50% |
| Range of proportion of funding from this source | 25-90% | 20-90% | 10-80% | 30% |  |

The most common combination of funders was CCG and NHS Trust, followed by NHS Trust and charity. Colleges with funding from a CCG or NHS Trust were most likely to receive half of their funding from this source, however this was variable, with some colleges reporting up to 90% of funding from either a CCG or NHS Trust.

The funders contributing to each of the seven colleges with three or more funding sources are summarised in Figure S6.1.

**Figure S6.1: Funding sources for colleges (n=7) reporting three or more funders**

Among the colleges with three or more funders, NHS Trusts or CCGs were still a major source of funding, providing at least 50% of funding for five of the seven colleges. All of the colleges with three of more funders had some element of charity funding, typically around 30%.

For the 48 colleges who reported their total budget and breakdown by funder, their total combined annual budget was £11,231,332. The breakdown of this budget by funder group, with NHS Trust and CCG combined into a single ‘NHS funder’ category, is shown in Figure S6.2.

**Figure S6.2: Proportion of combined budgets from 48 colleges by funder**


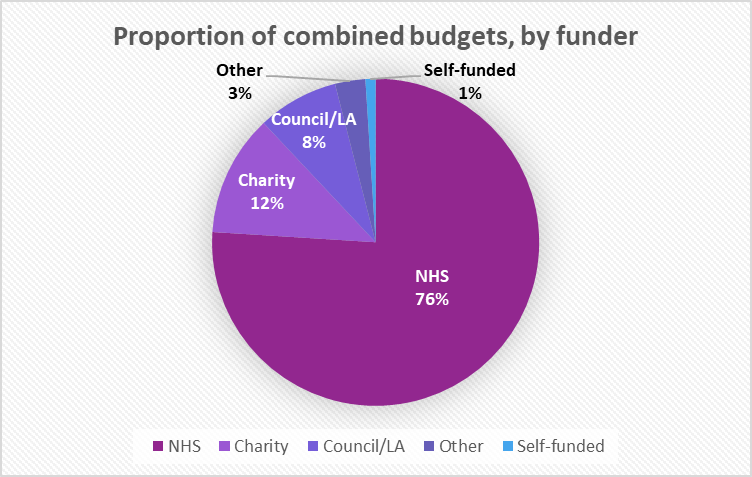


The NHS provides by far the largest proportion of the overall funding (76%) followed by charities (12%) and councils/local authorities (8%).

**Breakdown of spending**

In the survey we asked about costs across a range of different categories, however these were not exhaustive and so the costs across the categories do not add up to the total budgets summarised in Table S6.4.

**Table S6.4: Proportion of budget spent by category**

|  | **Number of colleges with a response** | **Mean (SD)** | **Median (IQR)** | **Range** |
| --- | --- | --- | --- | --- |
| Rent | 45 | 4.7% (8.0) | 0.1% (0.0%-6.1%) | 0.0% to 28.6% |
| Technology | 44 | 2.4% (4.1) | 0.7% (0.0%-2.8%) | 0.0% to 20.0% |
| Staff * | 30 | 76.4% (18.0) | 83.3% (66.7%-88.9%) | 31.2% to 97.1% |
| Staff training | 46 | 0.5% (1.0) | 0% (0.0%-0.9%) | 0.0% to 4.8% |

*six colleges have higher staff budgets than the total budget they reported – these have been excluded from this table

Other than staff budget, at least 25% of the colleges reported not having any costs for each of the other categories which is reflected in the median values which are less than 1%. A number of the colleges reported that staff training budgets were already incorporated within other budgets therefore the data collected may not be an accurate reflection of the amount of money spent on staff training.

**Staffing profiles**

For context, the NHS uses Agenda for Change pay bands referring to the following pay scales:

- Band 1 - <£18,000/year (<£9/hour)
- Band 2 – £18,001-19,800/year (£9-<10/hour)
- Band 3 – £19,801-21,900/year (£10-<11/hour)
- Band 4 – £21,901-24,900/year (£11-<13/hour)
- Band 5 – £24,901-31,400/year (£13-<16/hour)
- Band 6 – £31,401-38,900/year (£16-<20/hour)
- Band 7 – £38,901-£45,800/year (£20-<24/hour)
- Band 8a – £45,801-53,200/year (£24-<27/hour)
- Band 8b –£53,201-63,800/year (£27-<33/hour)
- Band 8c - £63,801-76,000/year (£33-<39/hour)
- Band 8d - £76,001-91,000/year (£39-<47/hour)
- Band 9 - £91,001+/year (£47+/hour)

The NHS pay banding and whole time equivalent (WTE) of core college staff are summarised in Table S6.5.

**Table S6.5: Core staff roles, pay bands, and total WTE**

| **Role** | **Range of pay bands** | **Total WTE**  median (IQR) [mean; SD] |
| --- | --- | --- |
| Managerial and “lead” roles | Managers only: Bands 5 to 9  Manager / lead roles: Bands 3 to 9 | 1.0 (1.0-1.6)  [1.4; 1.0] |
| Co-ordinators | Bands 4 to 5 | 1.5 (1.0-3.0)** |
| Administrators | Bands 1 to 5 | 1.0 (1.0-2.0)  [1.6; 1.3] |
| Peer trainers/facilitators | Bands 3 to 6 | 2.0 (1.0-4.5)  [3.6; 4.0] |
| Professional (non-peer) trainers / facilitators* | Bands 4 to 7 | 2.0 (1.0-3.0)  [2.1; 1.3] |
| Marketing / communications | Bands 4 to 5 | 0.8 (0.1-0.8)** |
| Other roles | Bands 2 to 6 | 1.0 (0.6-2.4)** |

*Trainers/facilitators with professional background i.e. not employed as “peers”

**mean and SD not calculated when fewer than 10 colleges reported having the role

The roles with the highest WTE are peer trainers and professional trainers. The salary bands are highly variable within many of the roles. The interquartile range and standard deviation around the WTE is largest for peer trainers which suggests there is a lot of variability across the colleges.

Fifteen colleges reported having unpaid or voluntary roles which were core to the college. The number of unpaid work hours per week in these colleges was median 28 hours (IQR 15-80) per college, with a mean of 41 hours (SD 35) and a range of 1 hour to 118 hours. The college with 118 hours per week of unpaid core roles reported that this was predominantly for workshop facilitators and learner support staff. The median budget in the colleges with unpaid core roles was £105,000 (IQR £37,000-£222,412) which was approximately half of the median budget for the colleges who did not report unpaid core roles (£206,000; IQR £150,000-338,000).

Unpaid roles reported by exactly one college were board member, cleaner, IT support, librarian, vice chair and video crew. Unpaid roles reported by more than one college are summarised in Table S6.6.

**Table S6.6 – Unpaid core staff at recovery colleges**

| **Role** | **Colleges with this role**  n | **Hours worked per week**  Median (IQR) [range] |
| --- | --- | --- |
| Trainers / facilitators | 8 | 20.5 (8.0-48.0)  [2-75] |
| Peer supporters / experts by experience | 6 | 10.5 (1.3-41.0)  [0.5-42] |
| Unspecified “volunteer” | 6 | 22.5 (4.3-52.0)  [0.5-60] |
| Administrators | 4 | 5.0 (1.0-9.0)  [1-20] |

The healthcare professionals who deliver occasional sessions at 68 recovery colleges are summarised in Table S6.7.

**Table S6.7: Healthcare professionals involvement in delivering sessions at Recovery Colleges (n=68)**

| **Role** | **Does the college have staff involved from this role?** | **Does the college pay directly for the role?** | **Hours worked per year**  Median (IQR) [mean; SD] |
| --- | --- | --- | --- |
| **Main clinical roles** |  |  |  |
| Occupational Therapist | Yes – 41%  No – 59% | Yes – 10%  No – 90% | 45 (20-109)  [308; 706]  n=28 |
| Psychologist | Yes – 39%  No – 61% | No – 100% | 20 (6-40)  [30; 33]  n=30 |
| Nurse | Yes – 34%  No – 66% | Yes – 4%  No – 96% | 30 (20-75)  [164; 423]  n=23 |
| Allied Health Professional | Yes – 32%  No – 68% | No – 100% | 22 (4-33)  [26; 25]  n=24 |
| Psychiatrist | Yes – 19%  No – 81% | No – 100% | 8 (2-23)  [21; 30]  n=20 |
| **Other roles** |  |  |  |
|  | **Number of colleges with this role** | **Does the college pay directly for the role?** | **Hours worked per year**  Median (IQR) [range] |
| Dietician / Nutritionist | 5 | Yes – 1  No – 3  Unknown - 1 | 12 (5-30)  [2-35] |
| Pharmacist | 2 | Yes – 1  No – 1 | [12-20] |
| Assistant Psychologist / Psychological Wellbeing Practitioner / CBT therapist | 5 | Yes – 3  No – 2 | 30 (20-40)  [20-780] |
| Social worker | 2 | No – 2 | [10-20] |

Of the main roles, the most common was Occupational Therapists who deliver sessions in 41% of the colleges and had the highest median number of hours worked per year. This was closely followed by Psychologists, who deliver sessions in 39% of colleges. Just under one-fifth (19%) of the colleges reported having sessions delivered by Psychiatrists, who also deliver the lowest median numbers of hours per year at the colleges. Colleges do not pay directly for input from professionals in almost all cases. The other roles reported by more than one college are also shown in Table S6.7. There was also one college who reported that a GP delivers at least one session per year.

**Business Cases**

Of the 60 colleges who answered the question about whether they had paid for or submitted any recent business cases for important new staff roles or facilities, 21 (35%) reported that they had done so. Some colleges reported business cases in relation to more than one area. The areas which the budget cases related to are summarised in Table S6.8.

**Table S6.8: Recent business cases reported by recovery colleges**

| **Area of budget/spending** | **Number of colleges reporting a business case in this area** |
| --- | --- |
| Staff/facilitators | 10 |
| Technology/online resources/equipment | 5 |
| Expansion of recovery college | 4 |
| Other | 5 |

By far the most common area for business cases was around staff budget, with almost half of business cases relating to this.

**Impact of COVID-19 on budgets**

Of the 60 colleges who answered the question about whether the COVID-19 pandemic had an impact on their budget or spending, 39 (65%) reported that it had. Specific impacts which were commonly reported in the free text responses are shown in Table S6.9.

**Table S6.9: Common impacts of COVID-19 on spending**

|  | **Number of colleges reporting this impact** |
| --- | --- |
| **Increases in spending** |  |
| Technology / services to enable online provision of courses | 14 |
| **Decreases in spending** |  |
| Venue hire | 14 |
| Printing / stationery | 3 |
| Transport / travel | 4 |
| Not specified / general reduction in costs | 3 |

The majority of colleges reported moving to online provision, which for many was associated with decreased venue hire costs and increased costs for technology and online services. However, three colleges reported continuing with face-to-face provision which was associated with increased costs due to the pandemic, including hiring larger rooms to allow for social distancing, personal protective equipment, increased printing costs for workbooks, wipeable furniture, and setting up ‘outdoor learning’ courses. One college reported that the pandemic had made it more difficult to apply for funding for their college, whereas three colleges reported being able to access additional sources of funding, for example in relation to setting up online learning or via the government’s COVID relief fund. One college reported having a reduction in staff costs as a result of delivering fewer courses, and another college reported having difficulties in recruiting peer trainers during the pandemic.
